# The comparative responsiveness of Hospital Universitario Princesa Index and other composite indices for assessing rheumatoid arthritis activity

**DOI:** 10.1371/journal.pone.0214717

**Published:** 2019-04-10

**Authors:** Isidoro González-Álvaro, Isabel Castrejón, Loreto Carmona, on behalf of ACT-RAY, PROAR and EMECAR study groups

**Affiliations:** 1 Rheumatology Service, Hospital Universitario La Princesa, IIS-IP, Madrid, Spain; 2 Division of Rheumatology, Rush University Medical Center, Chicago, Illinois, United States of America; 3 Instituto de Salud Musculoesquelética (InMusc), Madrid, Spain; University of Tasmania, AUSTRALIA

## Abstract

**Objective:**

To evaluate the responsiveness in terms of correlation of the Hospital Universitario La Princesa Index (HUPI) comparatively to the traditional composite indices used to assess disease activity in rheumatoid arthritis (RA), and to compare the performance of HUPI-based response criteria with that of the EULAR response criteria.

**Methods:**

Secondary data analysis from the following studies: ACT-RAY (clinical trial), PROAR (early RA cohort) and EMECAR (pre-biologic era long term RA cohort). Responsiveness was evaluated by: 1) comparing change from baseline (Δ) of HUPI with Δ in other scores by calculating correlation coefficients; 2) calculating standardised effect sizes. The accuracy of response by HUPI and by EULAR criteria was analyzed using linear regressions in which the dependent variable was change in global assessment by physician (ΔGDA-Phy).

**Results:**

ΔHUPI correlation with change in all other indices ranged from 0.387 to 0.791); HUPI’s standardized effect size was larger than those from the other indices in each database used. In ACT-RAY, depending on visit, between 65 and 80% of patients were equally classified by HUPI and EULAR response criteria. However, HUPI criteria were slightly more stringent, with higher percentage of patients classified as non-responder, especially at early visits. HUPI response criteria showed a slightly higher accuracy than EULAR response criteria when using ΔGDA-Phy as gold standard.

**Conclusion:**

HUPI shows good responsiveness in terms of correlation in each studied scenario (clinical trial, early RA cohort, and established RA cohort). Response criteria by HUPI seem more stringent than EULAR’s.

## Introduction

Objective evaluation of disease activity in rheumatoid arthritis (RA) has become a keystone of disease management. Composite indices measuring disease activity have allowed implementing treat-to-target and tight-control strategies, both contributing the most to the improvement in RA outcome achieved in the last 15 years. The most frequently used indices to evaluate disease activity among rheumatologists are the Disease Activity Score (DAS28) [calculated with C-reactive protein (CRP) or with sedimentation rate (ESR)] and the Simplified Disease Activity Index (SDAI), since they have been widely validated, are endorsed by ACR and EULAR, and are commonly used to assess therapeutic response in clinical trials [1–5]. In addition, the Clinical Disease Activity Index (CDAI) is being increasingly used, as it is easier to calculate than the previous ones, despite limited validation.

However, during the last 10 years, a fair amount of evidence suggested that both, DAS28 and SDAI, present a gender bias, derived from differences between men and women in terms of pain perception and levels of erythrocyte sedimentation rate (ESR)[6–10]. Using these indices, the implementation of T2T strategy would be biased, leading to over-treatment in women, or under-treatment of men. This may lead to excess risk of adverse events in women or lower odds to achieve real disease control in men. In addition, assessment of response to treatment in clinical trials might also be biased [11].

The Hospital Universitario La Princesa Index (HUPI) was developed to avoid a gender bias in the assessment of RA disease activity by adjusting the contribution of tender joint counts and ESR by sex [12]. An additional advantage of HUPI is that it can be calculated with ESR, CRP, or both acute phase reactants (APR), producing an almost identical score and avoiding missing data in longitudinal studies [12, 13].

HUPI was initially developed and validated in PEARL, a longitudinal observational study nested in an early arthritis register, and is calculated as the sum of four variables (graded 0–3 according to their quartile distribution in the PEARL population [see S1 Table]): 28 tender and swollen joint counts, global disease assessment by patient and APR [13]. When both ESR and CRP are used to calculate the index, the average of their scores in S1 Table is used to calculate HUPI. Thus, the index ranges from 0 to 12, and its cut-offs for remission/low disease activity, low/moderate and moderate/high disease activity are 2, 5 and 9 respectively [13]. HUPI may have a “ceiling effect”, especially in groups of patients with very high disease activity, such as those included in clinical trials.

The objective of this study was to evaluate the responsiveness of the HUPI, in parallel to that of the classical indices—DAS28-ESR, DAS28-CRP, SDAI and CDAI—, in terms of correlation, using data from three cohorts. Furthermore, the performance of HUPI-based response criteria was compared with that of EULAR response criteria.

## Patients and methods

### Patients

As mentioned, HUPI was developed in PEARL, an early arthritis register from Madrid (Spain)[12]. In the present study, we evaluate it in other RA populations, including RA from other countries. HUPI was evaluated in three different scenarios: a) an international clinical trial, the ACT-RAY—very high disease activity at baseline, homogeneous intervention, strict follow-up and patients enrolled in different countries; b) an early arthritis population (PROAR) in which sensitivity to change may be tested in a setting different from the early arthritis population used to develop and validate HUPI; and c) a long term prevalent RA population engaged in the pre-biologic era (EMECAR).

#### The ACT-RAY clinical trial

ACT-RAY is a 2-year double-blind clinical trial (NCT00810199, EudraCT No 2008-001847-20) designed to evaluate the efficacy and safety of tocilizumab plus methotrexate or tocilizumab plus placebo in patients with persistent active disease despite methotrexate monotherapy. Inclusion criteria for ACT-RAY were RA classification according to 1987 ACR criteria [14], DAS28>4.4, and erosive disease, as described previously [15]. Data collected included demographics, RA characteristics, as well as baseline and 4-weekly clinical and laboratory data necessary to calculate DAS28-ESR, DAS28-CRP, SDAI, CDAI and HUPI [15, 16].

Since no relevant differences in clinical response were reported between patients treated with tocilizumab in monotherapy or in combination with methotrexate [15], we included patients’ data irrespective of their allocation group. Considering that after the first year, patients in ACT-RAY were allocated into four different T2T strategies [16], for the present study the analysis was performed only with data from baseline and 12, 24 and 52 weeks visits.

#### The PROAR cohort

PROAR was a longitudinal multicenter study including 5 consecutive patients from 34 Rheumatology Units in Spain. Patients were included if presented at least one swollen joint for less than a year, irrespective of fulfilling 1987 ACR criteria [14]. At baseline, patients should be naïve for disease modifying anti-rheumatoid drugs (DMARDs) or glucocorticosteroids. Evidence of infectious arthritis or crystal arthritis were considered exclusion criteria [17]. Follow-up was 5 years, from January 2001 to December 2006.

Data collection included all variables needed to calculate DAS28-ESR, DAS28-CRP, SDAI, CDAI and HUPI at baseline and at each 6-monthly visits [17]. For the present study only patients fulfilling the 1987 ACR RA criteria along the follow-up were included. Most of these patients started treatment with DMARDs at the beginning of follow-up (S2 Table). Therefore, for the responsiveness analysis, baseline and 6 months visits were analyzed.

#### The EMECAR cohort

EMECAR was a prospective longitudinal cohort of prevalent RA patients fulfilling 1987 ACR criteria [14] selected by random sampling in 34 Rheumatology Units from Spain. Follow-up took place from November 1999 to December 2004 with yearly visits. EMECAR database includes the required variables to calculate DAS28-ESR and HUPI, but not DAS28-CRP, SDAI or CDAI, since global disease assessment by physician (GDA-Phy) was not collected and C-reactive protein (CRP) values provided limited reliability. A detailed description of the EMECAR cohort has been published previously [18].

At baseline, no patient was under treatment with a TNF-antagonist or leflunomide. During 4 years of follow-up, 27% of patient started, at least, one of these treatments. As we have previously described, improvement along the follow-up in this long term RA population was limited [18]. However, since HUPI was developed in patients with early arthritis, we included information about EMECAR in order to compare the performance of HUPI compared to DAS28 in long standing disease.

### Ethical statement

This is a secondary analysis of anonymized data from patients included in ACT-RAY, EMECAR and PROAR studies. ACT-RAY clinical trial was approved by the Research Ethical Committee (REC) of all centers included in the study (see Acknoledgement section “Group ACT-RAY”). EMECAR study was approved by the REC of Hospital Universitario La Princesa and this approval was accepted by all centers included in the study (see Acknoledgement section “Group EMECAR”). PROAR study was approved by the REC of Hospital Universitario La Princesa and this approval was accepted by all centers included in the study (see Acknoledgement section “Group PROAR”).

ACT-RAY, PROAR and EMECAR studies were conducted according to the principles expressed in the Helsinki Declaration of 1983. All patients signed the respective written consent before study entry [15, 17, 18].

### Statistical analysis

We used STATA 12.0 for Windows (StataCorp LP, College Station, TX). To describe the three populations, means and standard deviation (SD), medians and interquartile range (IQR), as well as absolute and relative frequencies were used, depending on the distribution of variables.

The external responsiveness of HUPI was evaluated as recommended by Husted et al in three different populations[19]. We used Pearson correlation coefficient to describe how changes from baseline (ΔHUPI) to different follow-up visits (ACT-RAY visits 12, 24 or 52 weeks; PROAR visit 6 months; EMECAR visit 4 years) correlated with corresponding changes in the values of global disease activity assessed by patient (ΔGDA-Pat), ΔGDA-Phy, ΔDAS28-ESR, ΔDAS28-CRP. Spearman correlation was used with ΔSDAI and ΔCDAI, since the values of these indices do not follow a normal distribution. Internal responsiveness was also evaluated using standardized effect size (SES) calculated with MS Excell 2007 for Windows as the mean difference between baseline and each previously mentioned time points divided by the pooled standard deviation, as described by Hedges and Olkin [20, 21].

We evaluated how HUPI-based response criteria[13] behave in comparison to EULAR response criteria[22] using data from ACT-RAY. First, we tabulated the response with each set of response criteria and cross-tabulated them. To determine the accuracy of both response criteria, we used the percentage of correctly classified patients from the best fitted models with ΔGDA-Phy as external criterion. ΔGDA-Phy was used to avoid circularity, since neither HUPI nor DAS28 include this variable in their computation. Linear regression models using generalized linear solutions (Stata’s glm command with the default option) were performed with ΔGDA-Phy (from baseline to different time points) as dependent variable and HUPI-based and EULAR response criteria as categorical variables. Beta coefficients with 95% confidence intervals (95%CI) for “Moderate” and “Good response” by either definition were reported, along with the Akaike information criteria (AIC) from each model (S6 and S7 Tables). The later allow us to identify the best model; given two different regression models fitted on the same data, the model with the smallest AIC value is considered the best [23].

## Results

### Assessment of disease activity with different indices in three different populations

Table 1 shows a description of the three study populations. In all three, about 75% of patients were women and mean age at baseline ranged from 53 to 61 years. As part of the inclusion criteria, patients from the early arthritis cohort had the disease for less than a year in contrast with about 8 years in ACT-RAY and EMECAR. As expected, patients from the clinical trial showed the highest baseline disease activity and disability, EMECAR patients showed mid values, and those from PROAR showed the lowest scores of disease activity and disability (Table 1).

**Table 1:** Baseline characteristics of the populations.

	ACT-RAY (n = 556)	PROAR (n = 160)	EMECAR (n = 789)
Female gender, n (%)	446 (80.0)	113 (70.6)	568 (72.0)
Age, median [IQR]	54 [46–62.5]	54 [44–68]	64 [54–71]
Disease duration, median [IQR] (years)	8.3±8.2*	0.37 [0.21–0.63]	8.7 [4.1–12.6]
Smoker, n (%)	200 (36.0)	60 (37.5)	274 (35.4)
RF positive, n (%)	N.A.	47 (34.6)	578 (74.6)
ACPA positive, n (%)	N.A.	76 (47.8)	N.A.
HAQ, median [IQR]	1.5 [1–1.875]	0.5 [0–1.125]	1.125 [0.5–1.875]
HUPI, mean ± SD	10.9 ± 1.3	5.3 ± 3.1	6.5 ± 3.0
DAS28, mean ± SD	6.34 ± 0.99	3.84 ± 1.45	4.25 ± 1.40
DAS28-CRP, mean ± SD	6.44 ± 1.03	3.59 ± 1.33	N.A.
SDAI, median [IQR]	44.3 [34.1–55.5]	13.9 [6.0–21.7]	N.A.
CDAI, median [IQR]	38.0 [28.8–47.2]	11.9 [4.6–20.4]	N.A.

Abbreviations: n, number; IQR, interquartile range; SD, standard deviation; DO, disease onset; N.A., not available; RF, rheumatoid factor; ACPA anti-citrullinated protein antibodies; HAQ, health assessment questionnaire; HUPI, Hospital Universitario La Princesa Index; DAS28, disease activity score calculated with erythrocyte sedimentation rate and 28 joint counts; DAS28-CRP, disease activity score calculated with C-reactive protein and 28 joint counts; SDAI, simplified disease activity index; CDAI, clinical disease activity index.

*IQR of disease duration was not available for ACT-RAY patients.

As a result of HUPI allowing calculation from CRP or ESR, whichever available at the study visit—a strategy to minimize missing data—, the HUPI was calculated in more visits than the other indices in the three populations: 99.8% of visits in ACT-RAY; 96.7% in PROAR; and 92.3% in EMECAR, with the only exception of CDAI in PROAR: 98.6% (S3 Table).

**Fig 1:**
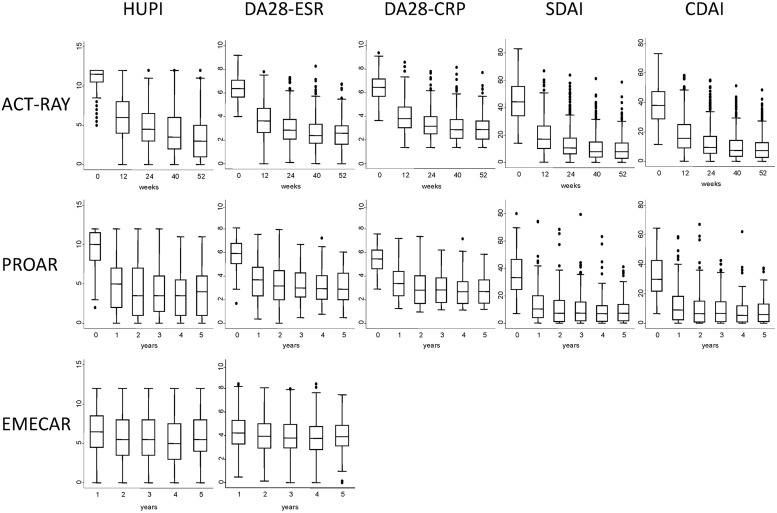
Evolution of disease activity during follow-up of the ACT-RAY, PROAR and EMECAR patients assessed with different indices. Data are presented as interquartile range (p75 upper edge, p25 lower edge, p50 midline in the box), p95 (line above the box) and p5 (line below the box). Dots represent outliers.

In patients from ACT-RAY, baseline HUPI values show a “ceiling effect” with more than 40% of patients at the highest score of the index (12 units; upper left panel in Fig 1). The remaining indices did not show this effect, with less of 5% at the highest value of SDAI and no patient at the highest score of DAS28-ESR, DAS28-CRP and CDAI (remaining panels in Fig 1). All indices showed improvement of disease activity after starting treatment with tocilizumab (Fig 1).

Patients from PROAR and EMECAR showed lower disease activity at baseline, so no “ceiling effect” was observed in HUPI (Fig 1 left panels of mid and lower row). Disease activity improvement with all indices was observed in PROAR after starting DMARD treatment in this early arthritis population (Fig 1 mid row). Limited improvement was observed in EMECAR (Fig 1 lower row).

### Responsiveness of HUPI versus traditional indices

**Fig 2:**
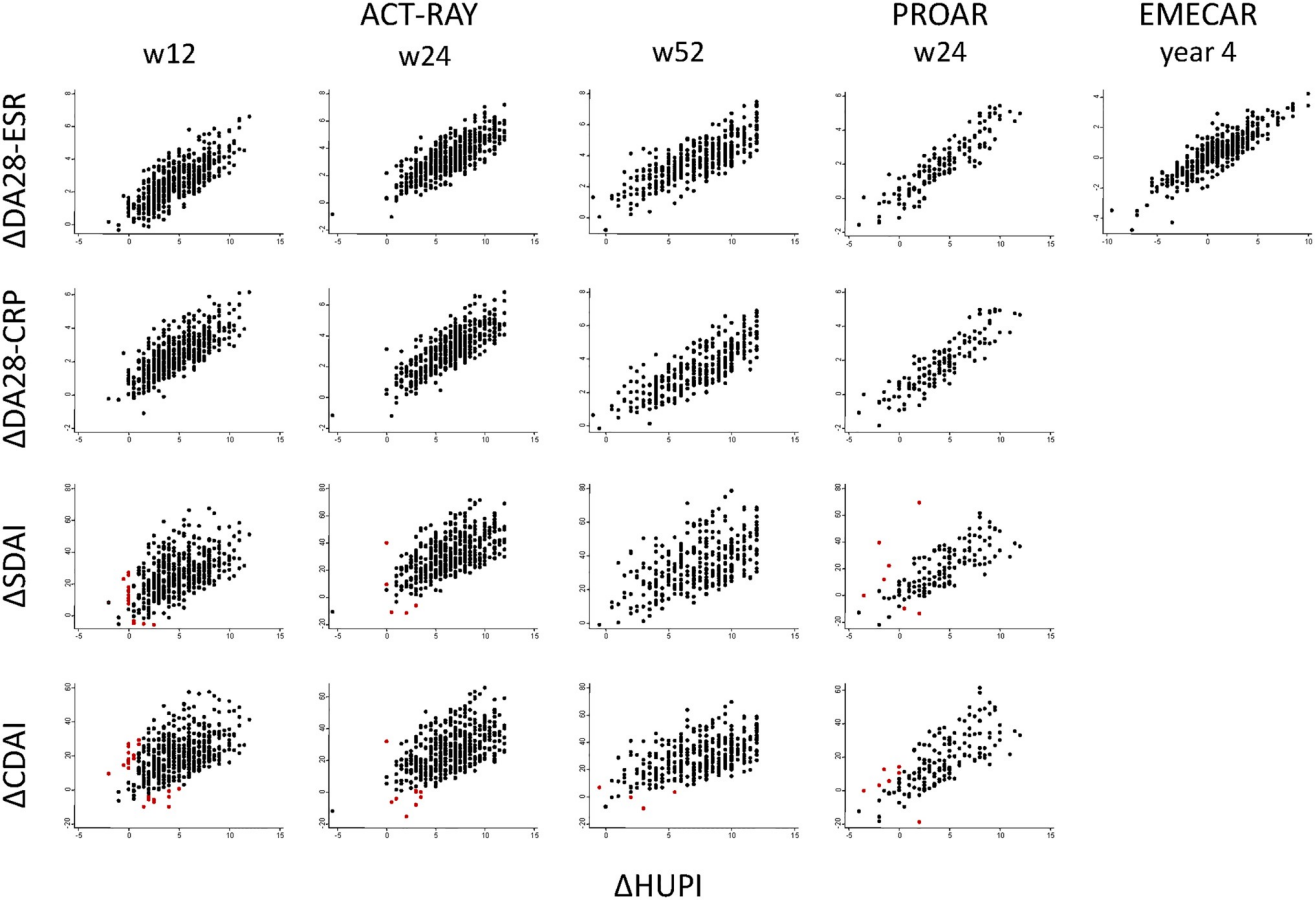
External responsiveness of HUPI (ΔHUPI) compared with improvement measured with other variables commonly used to assess clinical improvement in rheumatoid arthritis. Red dots represent values in which there was clear discordance between ΔHUPI and other measuments of improvement. The correlation coefficients are shown at Table 2.

Despite its baseline “ceiling effect” in the ACT-RAY clinical trial, the change in HUPI score at week 12 had a good correlation with ΔGDA-Pat and slightly lower with ΔGDA-Phy. Consequently, the correlation was very good with ΔDAS28 either with ESR or CRP, and slightly lower with ΔSDAI or ΔCDAI (Table 2 and Fig 2).

**Table 2:** External responsiveness of HUPI compared with improvement measured with other variables commonly used to assess response in rheumatoid arthritis.

	ACT-RAY			PROAR		EMECAR	
	w12 (n = 491)	w24 (n = 475)	w52 (n = 340)	w24 (n = 136)	w52 (n = 129)	Y2 (n = 560)	Y4 (n = 440)
**ΔGDA-Pat**	0.50 (0.43–0.56)	0.58 (0.52–0.63)	0.62 (0.55–0.68)	0.60 (0.48–0.70)	0.66 (0.55–0.75)	0.51 (0.45–0.57)	0.51 (0.44–0.78)
**ΔGDA-Phy**	0.39 (0.31–0.46)	0.52 (0.44–0.57)	0.53 (0.45–0.60)	0.65 (0.54–0.74)	0.63 (0.51–0.72)		
**ΔDAS28_ESR**	0.77 (0.73–0.80)	0.78 (0.74–0.81)	0.78 (0.73–0.82)	0.91 (0.88–0.94)	0.89 (0.85–0.92)	0.87 (0.85–0.89)	0.87 (0.84–0.89)
**ΔDAS28-CRP**	0.75 (0.71–0.79)	0.79 (0.75–0.81)	0.79 (0.75–0.83)	0.89 (0.85–0.92)	0.90 (0.86–0.93)		
**ΔSDAI**	0.52 (0.45–0.58)	0.56 (0.49–0.62)	0.51 (0.43–0.58)	0.75 (0.67–0.82)	0.78 (0.70–0.84)		
**ΔCDAI**	0.48 (0.41–0.55)	0.55 (0.48–0.61)	0.48 (0.39–0.56)	0.78 (0.70–0.84)	0.79 (0.72–0.85)		

GDA: global disease assessment; Pat: patient; Phy: physician; HUPI: Hospital Universitario La Princesa Index; DAS28_ESR or _CRP: disease activity score calculated with 28 joint counts and erythrosedimentation rate or C reactive protein; SDAI: simplified disease activity index; CDAI: clinical disease activity index; Data are shown as the correlation coefficient (95% confidence interval).

In ACT-RAY correlations tended to improve when comparing ΔHUPI from baseline and weeks 24 and 52 and the corresponding changes of the other variables (Table 2). Interestingly, Fig 2 shows that there were individual important disparities (red dots) between ΔHUPI and ΔSDAI or ΔCDAI.

**Fig 3:**
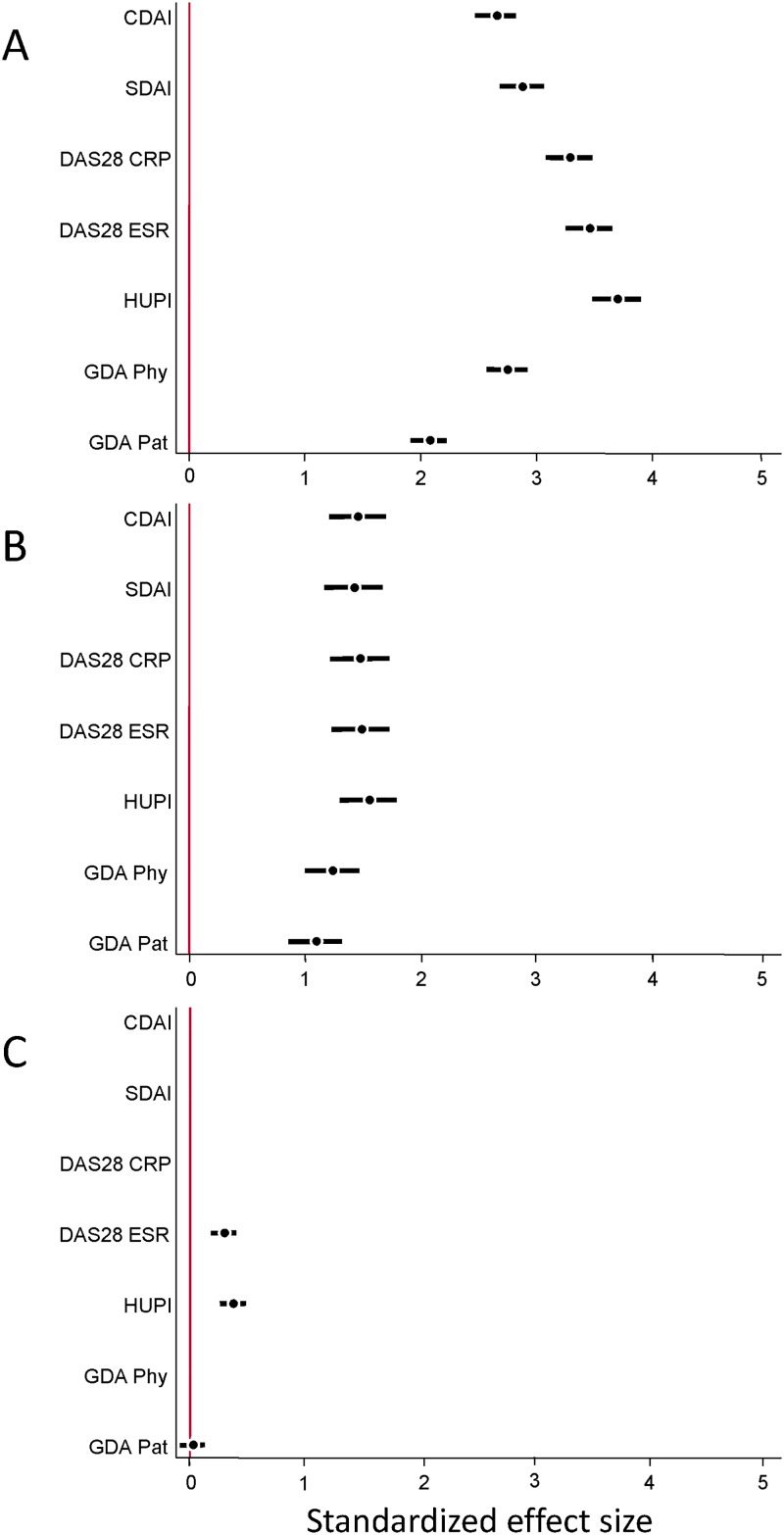
Internal responsiveness of different indices and variables used to assess disease activity in different cohorts. Data are shown as the standardized effect size (black circle) and its 95% confidence interval (black bar) at A) week 52 for ACT-RAY patients; B) week 24 for PROAR patients; and C) year 4 for EMECAR patients.

We hypothesized that this correlation would not be perfect since HUPI was specifically developed to avoid gender bias of DAS28 and SDAI. In addition, HUPI does not include GDA-Phy in its calculations. So, in order to be able to compare the respective sensitivity to change (Internal responsiveness), we calculated the SES for each variable at the three time points studied. The SES for HUPI was always the highest in the three populations at all times studied (Fig 3 and S4 Table). In addition, the 95% CI of HUPI’s SES did not overlap with those from GDA-Pat, GDA-Phy, SDAI and CDAI at any time in ACT-RAY (Fig 3A and S4 Table).

Similar findings were observed in patients from PROAR, although lower SES were observed since the baseline disease activity was lower than that of patients in ACT-RAY (Fig 1), with no significant differences across indices (Fig 3B and S4 Table).

On the other hand, limited disease activity improvement had been described with DAS28 in EMECAR[18] and the data with HUPI were consistent with these previous findings (Fig 3C and S4 Table).

### Comparison of EULAR response criteria and HUPI-based response criteria

**Fig 4:**
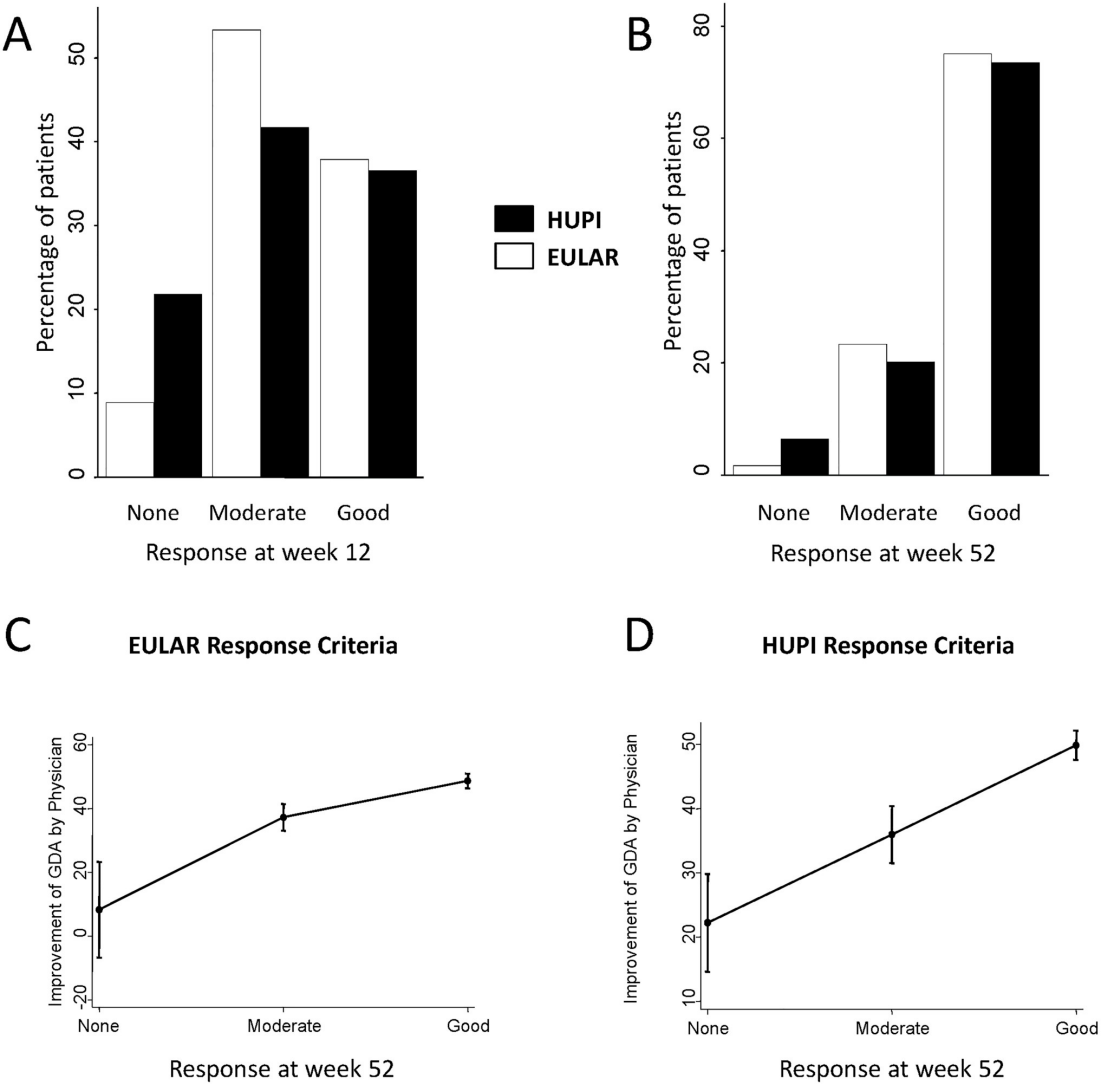
Comparison of EULAR and HUPI response criteria from baseline at different weeks of patients from ACT-RAY. A) Percentage of patients getting none, moderate or good response at week 12. B) Percentage of patients getting none, moderate or good response at week 52 (See S1 Fig panel A for information at week 24). C) Association of change in global disease assessment by physician (GDA-Phy) with the different categories of EULAR response (See S1 Fig panel B for information at weeks 12 and 24). D) Association of change in GDA-Phy with the different categories of HUPI response (See S1 Fig panel B for information at weeks 12 and 24). Data in panels C and D are shown as the predicted mean change in GDA-Phy with its 95% confidence interval for each category obtained from the linear regression models showed in S6 Table with the command *marginsplot* of Stata.

In ACT-RAY, it was possible to determine HUPI response in more patients than with EULAR response criteria either at week 12 (528 vs. 518), week 24 (509 vs. 503) or week 52 (418 vs. 412). The lower number of assessments with EULAR response was due to missing ESR, required to calculate DAS28. In addition, the proportion of patients with no response was higher with HUPI than with EULAR response criteria, although gradually being the proportions closer along the follow-up (Fig 4A and 4B and S1 Fig panel A). Similar findings were observed in PROAR and EMECAR (S2 Fig).

Table 3 shows that response to treatment was equally classified by both criteria in > 65% of patients at week 12 and it gradually increased to >80% of patients at week 52. Three patients were classified as good responders with EULAR response criteria and non-responders with HUPI at all three time-points, whereas 1 patient was classified as good responder with HUPI but non-responder with EULAR only at week 12. The characteristics of these patients are shown in S5 Table. In summary, 3 patients had high number of tender joints, but low number of swollen joints, at baseline that improved in terms of tender joints but neither in terms of swollen counts, nor APR or GDA-Pat. By contrast, the patient with no response by EULAR criteria but good response with HUPI was a female patient with extremely high number of tender joints that did not improve with treatment, whereas the remaining parameters were low at baseline and improved with treatment.

**Table 3:** Distribution of patients from ACT-RAY depending on clinical response assessed either by EULAR or HUPI criteria at different time-points.

	Week 12 (n = 518)			Week 24 (n = 503)			Week 52 (n = 412)		
EULAR	HUPI			HUPI			HUPI		
	None	Moderate	Good	None	Moderate	Good	None	Moderate	Good
**None**	40	5	***1***	11	3	***0***	5	2	***0***
**Moderate**	70	164	42	34	99	36	19	54	23
**Good**	***3***	47	146	***3***	43	274	***3***	26	280
**Same response**	67.6%			76.3%			82.3%		

Finally, to analyze which response set may be more accurate, we used as external criterion the ΔGDA-Phy from baseline to 12, 24 and 52 weeks. As shown in Fig 3C and S1 Fig panel B, in average an improvement of GDA-Phy≤20 was associated with no response at all time points for HUPI response criteria and at week 12 for EULAR-RC. In the following time-points EULAR no response tended to be associated with lower ΔGDA-Phy. Regarding moderate and good responses, by HUPI criteria the average improvement in ΔGDA-Phy tended to be more lineal, whereas by EULAR criteria, higher improvement in ΔGDA-Phy were needed to reach moderate response and then lower improvements were needed to reach good response with respect moderate response (Fig 2C and 2D and S2 Fig panel B). This can be appreciated with the beta coefficients of the linear correlation models, in which the Akaike information criteria was always lower for the models run with HUPI than with EULAR response, suggesting that the former were better fitted (S6 Table). Similar results were observed in the PROAR and EMECAR cohorts (S3 Fig and S7 Table).

## Discussion

HUPI was developed in an intent to provide a more accurate tool for assessing disease activity in patients with early RA and undifferentiated arthritis [12]. Validation is an ongoing process and new instruments like the HUPI need to be tested in different populations; therefore, we aimed to further validate HUPI by evaluating its responsiveness and the recently proposed HUPI-based response criteria [13]. This was particularly necessary in patients from clinical trials, whose baseline disease activity, as part of the general inclusion criteria, is usually very high. At present, there is no gold standard to assess disease activity in RA, nevertheless we used pooled indices of multiple measures that have been previously developed based on the Core Data Set proposed by Felson et al[24]. All in all, the data presented in this work showed that HUPI exhibits comparable responsiveness to that of DAS28 and better than SDAI and CDAI. In addition, our data suggest that HUPI-based response criteria are slightly more stringent than EULAR’s.

Baseline data from ACT-RAY have allowed confirming that in a clinical trial setting HUPI has a “ceiling effect”, likely due to its design. Remarkably, this ceiling effect was not detected in the other two cohorts more representative of patients seen in routine care. Nevertheless, in our opinion, patients with 5 or more swollen and tender joints and GDA-Pat higher than 50/100 and CRP levels higher 1 mg/dl show very high disease activity and need special therapeutic approaches irrespective of the magnitude of these variables above these limits.

Despite this “ceiling effect”, HUPI showed the largest sensitivity to change in all three populations, with SES superior to those of SDAI, CDAI and GDA, either by physician and patient. We recognize that SES is not the best statistic to report responsiveness, as it is only assessing internal responsiveness; however, it allows comparison across indices with varying range of values. In addition, similar results were reported using other methods when we described the index [12]. The poorer responsiveness of SDAI and CDAI may be a consequence of their design’s simplicity, leading to non-normally distributed variables with a highly spread range of values in moderate and high disease activity. This can also explain the disparities in response measurements showed at Fig 2.

On the other hand, since the responsiveness of HUPI is quite similar to that of DAS28, the response criteria based in both indices behave very similarly. Small differences have been detected, being HUPI slightly more stringent, with larger percentages of patients considered non-responders in ACT-RAY compared to percentages of EULAR response. These differences decreased along follow-up, although they were still detected at week 52, being the fast effect of tocilizumab on APR a possible explanation, since ESR is highly weighted in DAS28 [25]. Another possibility to explain this discrepancy is the tender joint count, that is also highly weighted in DAS28 and in HUPI is weighted differently by gender [12]. In this regard, it has been described that the presence of fibromyalgia can interfere with the assessment of disease activity with DAS28, since it impacts in the subjective components of the index, such as tender joint count[26].

Nevertheless, it is difficult to know whether being HUPI-based response more stringent than EULAR response may be an advantage or a disadvantage. In ACT-RAY, all patients were treated with tocilizumab plus placebo or methotrexate, showing no statistical differences between groups, but a statistical difference from baseline in both groups [15]. On these grounds, we considered patients from both groups experiencing a similar change; however, it was not our aim to evaluate treatment effect with any index. For this reason, we cannot determine whether HUPI-based response is as stringent in a “real” placebo group as in an active treatment group, nor whether it helps discriminating the effect of the drug.

## Conclusion

In summary, despite its “ceiling effect”, HUPI shows good responsiveness in all the scenarios tested. In addition, the response criteria based on this new index seems to be more stringent than the EULAR response criteria, although we need to deepen in the study of this characteristic to determine whether it could be more efficient to detect differences between placebo and active treatment.

## Supporting information

S1 Fig Comparison of EULAR and HUPI response criteria from baseline at different weeks of patients from ACT-RAY. A) Percentage of patients getting none, moderate or good response at week 24. B) Correlation of change in global disease assessment by physician (GDA-Phy) with the different categories of EULAR response and HUPI response at week 12 and week 24. Data in panels in section B are shown as the predicted mean change in GDA-Phy with its 95% confidence interval for each category obtained from the linear regression models showed in S6 Table.
(TIF)

S2 Fig Comparison of EULAR and HUPI response criteria from baseline at different weeks of patients from PROAR and EMECAR. A) Percentage of patients getting none, moderate or good response at month 6, month 12 and month 24 in PROAR. B) Percentage of patients getting none, moderate or good response at 4 years of follow-up in EMECAR.
(TIF)

S3 Fig Correlation of change in global disease assessment by physician (GDA-Phy) with the different categories of EULAR response and HUPI response in PROAR at months 6 (A), 12 (B) or 24 (C) and EMECAR at year 4^th^ (D). Data are shown as the predicted mean change in GDA-Phy with its 95% confidence interval for each category obtained from the linear regression models showed in S7 Table.
(TIF)

S1 Table Scoring of the variables used to calculate HUPI. (DOCX)

S2 Table DMARD prescription during follow-up in PROAR. (DOCX)

S3 Table Number of visits from the different cohorts in which it was possible to calculate each index. (DOCX)

S4 Table Standardized size effects (95% confidence interval) of changes in disease activity assessed with HUPI and several other commonly used to assess response in rheumatoid arthritis. (DOCX)

S5 Table Characteristics of patients with opposite classification with EULAR and HUPI response criteria. (DOCX)

S6 Table Accuracy of EULAR-RC and HUPI-RC assessed by their correlation with change in GDA-Phy between baseline and different visits of ACT-RAY. (DOCX)

S7 Table Accuracy of EULAR and HUPI-based response criteria assessed by their correlation with ΔGDA-Phy in PROAR and ΔGDA-Pat in EMECAR. (DOCX)

S1 File EMECAR data in longitudinal format. This Excell file includes in longitudinal format all data from EMECAR cohort used to develop the results shown in this paper.
(XLS)

S2 File EMECAR data in wide format. This Excell file includes in wide format all data from EMECAR cohort used to develop the results shown in this paper.
(XLS)

S3 File PROAR data in longitudinal format. This Excell file includes in longitudinal format all data from PROAR cohort used to develop the results shown in this paper.
(XLS)

S4 File PROAR data in wide format. This Excell file includes in wide format all data from PROAR cohort used to develop the results shown in this paper.
(XLS)
