# Supplementary figures and images for: The comparative responsiveness of Hospital Universitario Princesa Index and other composite indices for assessing rheumatoid arthritis activity
Source: PLoS One. 2019 Apr 10;14(4):e0214717. doi: 10.1371/journal.pone.0214717 (PMC6457549; doi:10.1371/journal.pone.0214717)

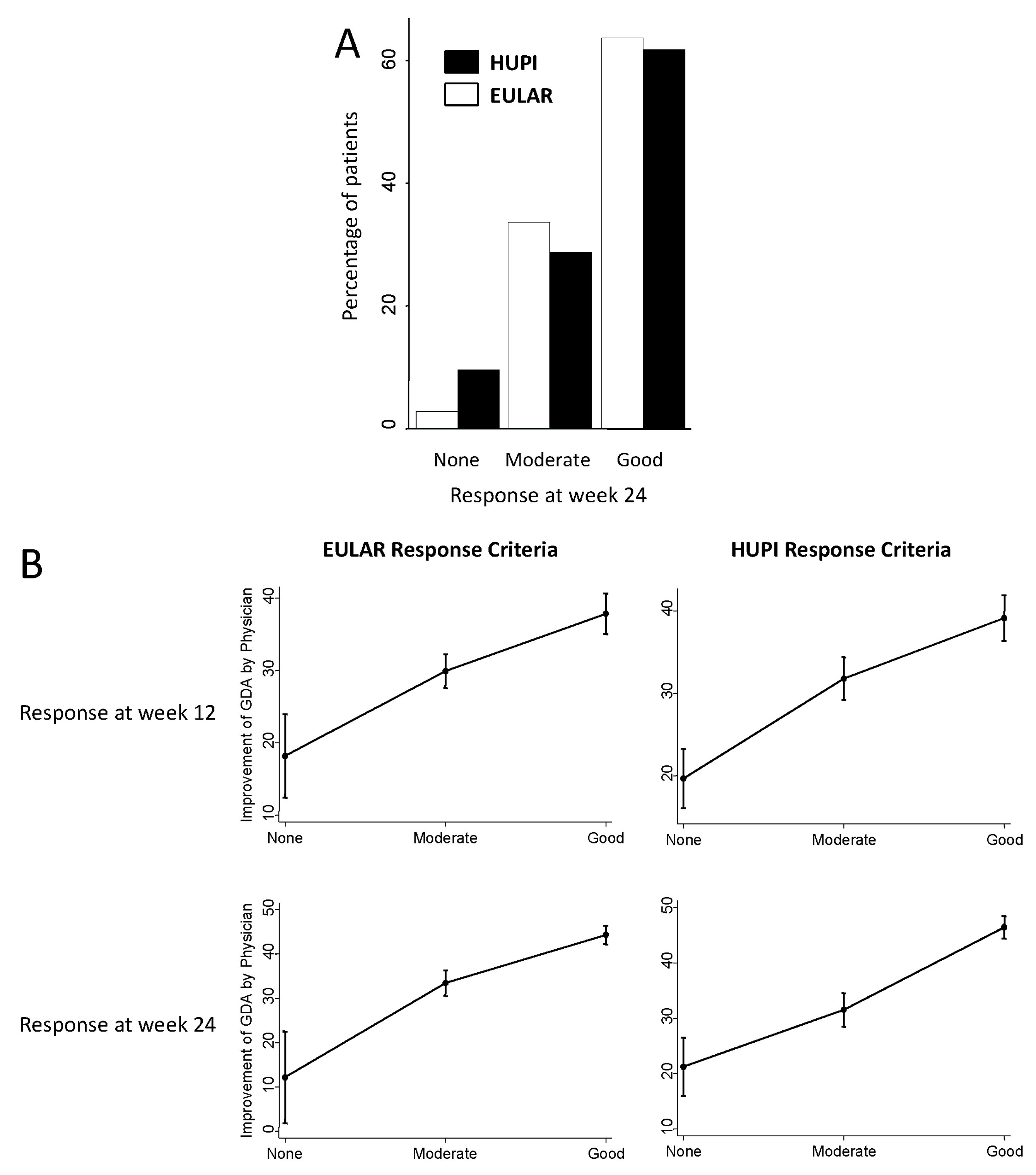

Supplement: S1 Fig — A) Percentage of patients getting none, moderate or good response at week 24. B) Correlation of change in global disease assessment by physician (GDA-Phy) with the different categories of EULAR response and HUPI response at week 12 and week 24. Data in panels in section B are shown as the predicted mean change in GDA-Phy with its 95% confidence interval for each category obtained from the linear regression models showed in S6 Table. (TIF) [file pone.0214717.s001.tif]

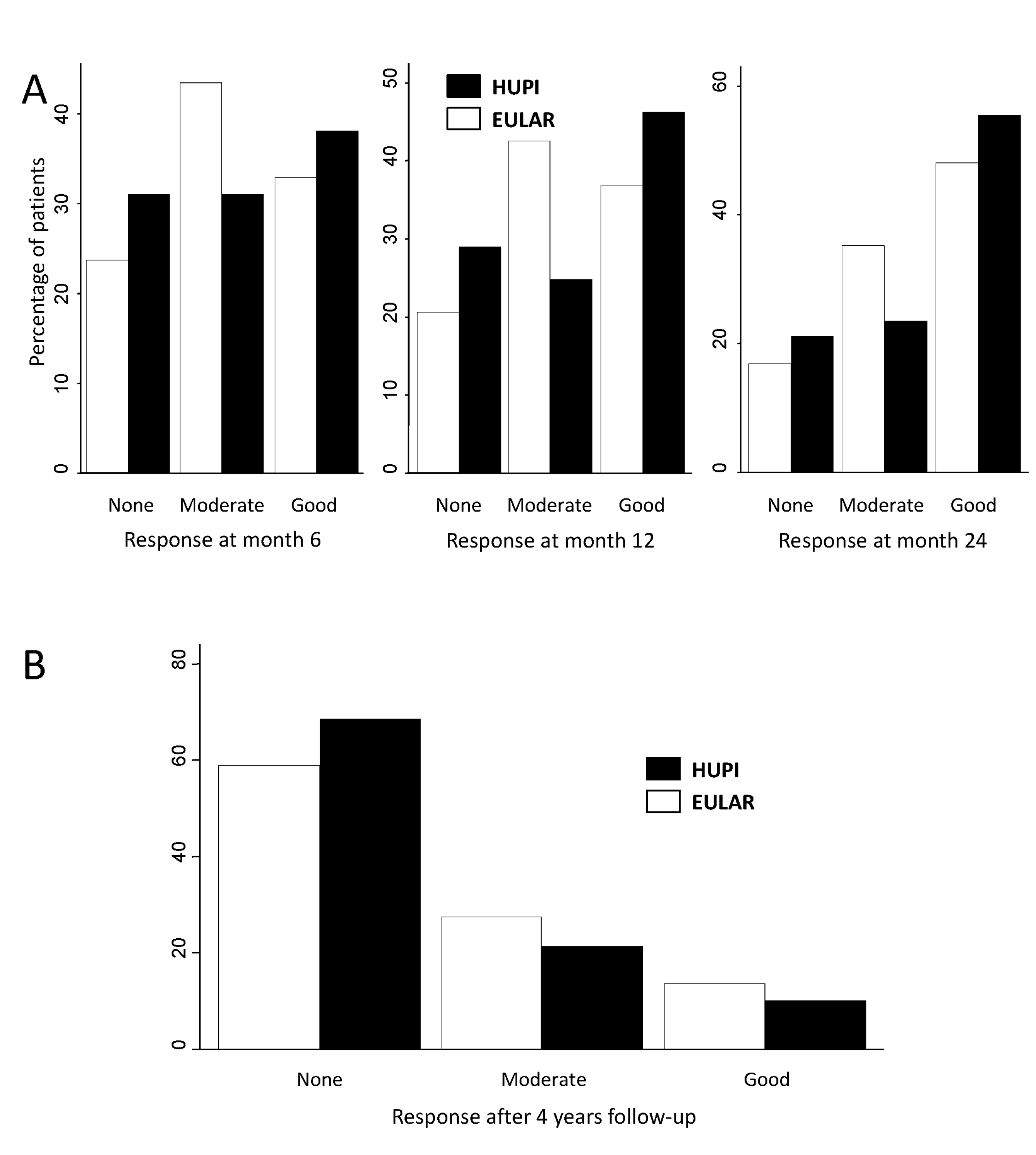

Supplement: S2 Fig — A) Percentage of patients getting none, moderate or good response at month 6, month 12 and month 24 in PROAR. B) Percentage of patients getting none, moderate or good response at 4 years of follow-up in EMECAR. (TIF) [file pone.0214717.s002.tif]

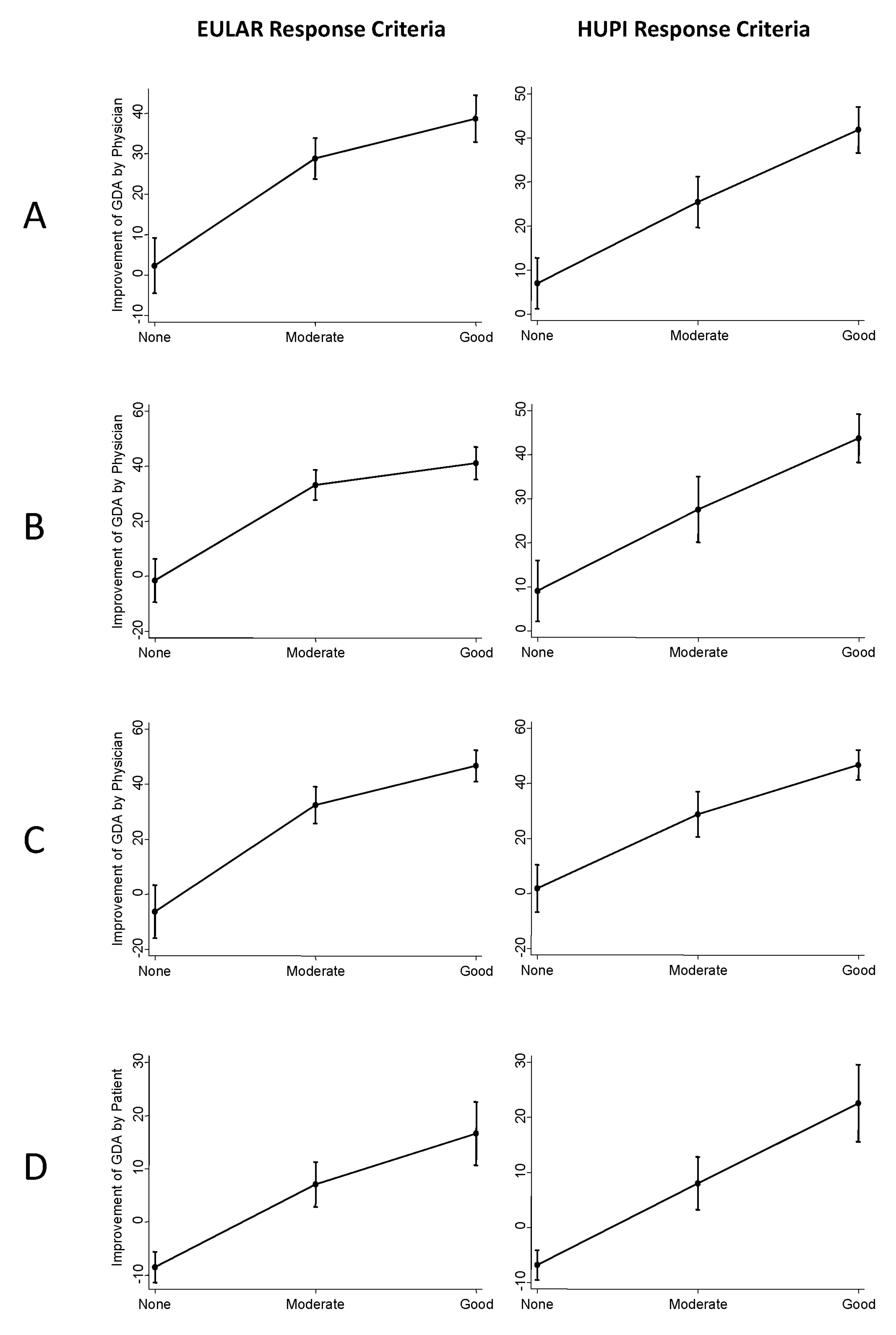

Supplement: S3 Fig — Data are shown as the predicted mean change in GDA-Phy with its 95% confidence interval for each category obtained from the linear regression models showed in S7 Table. (TIF) [file pone.0214717.s003.tif]
