# Supplementary material for: The comparative responsiveness of Hospital Universitario Princesa Index and other composite indices for assessing rheumatoid arthritis activity
Source: PLoS One. 2019 Apr 10;14(4):e0214717. doi: 10.1371/journal.pone.0214717 (PMC6457549; doi:10.1371/journal.pone.0214717)
Supplement: S1 Table — (DOCX) [file pone.0214717.s004.docx]

**S1 Table. Scoring of the variables used to calculate HUPI.**

This information was previously described at Gonzalez-Alvaro I, Castrejon I, Ortiz AM, Toledano E, Castaneda S, Garcia-Vadillo A, et al. Cut-Offs and Response Criteria for the Hospital Universitario La Princesa Index (HUPI) and Their Comparison to Widely-Used Indices of Disease Activity in Rheumatoid Arthritis. PLoS One. 2016;11(9):e0161727.
