# Supplementary material for: The comparative responsiveness of Hospital Universitario Princesa Index and other composite indices for assessing rheumatoid arthritis activity
Source: PLoS One. 2019 Apr 10;14(4):e0214717. doi: 10.1371/journal.pone.0214717 (PMC6457549; doi:10.1371/journal.pone.0214717)
Supplement: S2 Table — (DOCX) [file pone.0214717.s005.docx]

**S2 Table. DMARD prescription during follow-up in PROAR.**

|  | 6 months  (n=150) | 1^st^ year  (n=142) | 2^nd^ year  (n=113) | 3^rd^ year  (n=58) | 4^th^ year  (n=53) | 5^th^ year  (n=41) |
| --- | --- | --- | --- | --- | --- | --- |
| Methotrexate | 87 (58%) | 85 (59.9%) | 81 (71.7%) | 58 (100%) | 53 (100%) | 41 (100%) |
| Antimalarials | 26 (17.3%) | 30 (21.1%) | 30 (26.5%) | 19 (32.7%) | 14 (26.4%) | 10 (24.4%) |
| Gold salts | 13 (8.7%) | 9 (6.3%) | 5 (4.4%) | 2 (3.4%) | 2 (3.8%) | 1 (2.4%) |
| Sulphasalazine | 10 (6.7%) | 13 (9.1%) | 11 (9.7%) | 4 (6.9%) | 4 (7.5%) | 3 (7.3%) |
| Leflunomide | 7 (4.7%) | 11 (7.7%) | 15 (13.3%) | 17 (29.3%) | 17 (32.1%) | 14 (34.1%) |
| Ciclosporin A | 0 | 0 | 1 (0.8%) | 1 (1.7%) | 3 (5.7%) | 0 |
| DPA | 0 | 0 | 1 (0.8%) | 0 | 0 | 0 |
| TNF-blockers | 0 | 2 (1.4%) | 5 (4.4%) | 7 (12.1%) | 17 (32.1%) | 14 (34.1%) |
| Prednisone | 105 (70%) | 89 (62.7%) | 67 (59.3%) | 48 (82.7%) | 46 (86.8%) | 30 (73.2%) |

DPA: D-penicillamine.
