# Supplementary material for: The comparative responsiveness of Hospital Universitario Princesa Index and other composite indices for assessing rheumatoid arthritis activity
Source: PLoS One. 2019 Apr 10;14(4):e0214717. doi: 10.1371/journal.pone.0214717 (PMC6457549; doi:10.1371/journal.pone.0214717)
Supplement: S3 Table — (DOCX) [file pone.0214717.s006.docx]

**S3 Table**. **Number of visits from the different cohorts in which it was possible to calculate each index.**

|  | **ACT-RAY (8,210)** | **PROAR (n=1,315)** | **EMECAR (n=3,079)** |
| --- | --- | --- | --- |
| **HUPI**  n (mean ± SD) | 8,197 (5.2 ± 3.2) | 1,283 (5.1 ± 3.6) | 2,842 (5.9 ± 2.9) |
| Remission | 1,841 (22.4) | 370 (28.8) | 336 (11.8) |
| Low DA | 2,606 (31.8) | 323 (25.2) | 938 (33.0) |
| Moderate DA | 2,379 (29.1) | 336 (26.2) | 1,046 (36.8) |
| High DA | 1,371 (16.7) | 254 (19.8) | 522 (18.4) |
| **DAS28-ESR**  n (mean ± SD) | 8,089 (3.31 ± 1.6) | 1,268 (3.7 ± 1.7) | 2,755 (4.0 ± 1.4) |
| Remission | 2,964 (36.6) | 401 (31.6) | 553 (20.1) |
| Low DA | 1,384 (17.1) | 149 (11.8) | 415 (15.1) |
| Moderate DA | 2,579 (31.9) | 454 (35.8) | 1,274 (46.2) |
| High DA | 1,162 (14.4) | 264 (20.8) | 513 (18.6) |
| **DAS28-CRP**  n (mean ± SD) | 8,197 (3.6 ± 1.4) | 1,222 (3.4 ± 1.6) | N.A. |
| Remission | 1,576 (19.2) | 372 (30.4) |  |
| Low DA | 3,482 (42.5) | 382 (31.3) |  |
| Moderate DA | 1,645 (20.1) | 247 (20.2) |  |
| High DA | 1,494 (18.2) | 221 (18.1) |  |
| **SDAI**  n (mean ± SD) | 8,118 (16.6 ± 13.9) | 1,219 (15.0 ± 15.1) | N.A. |
| Remission | 1,021 (12.6) | 317 (26.0) |  |
| Low DA | 2,676 (33.0) | 301 (24.7) |  |
| Moderate DA | 2,773 (34.1) | 367 (30.1) |  |
| High DA | 1,648 (20.3) | 234 (19.2) |  |
| **CDAI**  n (mean ± SD) | 8,118 (14.9 ± 12.9) | 1,297 (13.3 ± 13.5) | N.A. |
| Remission | 1,111 (13.7) | 353 (27.2) |  |
| Low DA | 2,579 (31.8) | 315 (24.3) |  |
| Moderate DA | 2,550 (31.4) | 354 (27.3) |  |
| High DA | 1,878 (23.1) | 275 (21.2) |  |

Results are expressed in n (%) unless otherwise noted.

Abbreviations: n, number; SD, standard deviation; DA, disease activity; N.A., not available. HUPI, *Hospital Universitario La Princesa* Index; DAS28, disease activity score calculated with erythrocyte sedimentation rate and 28 joint counts; DAS28-CRP, disease activity score calculated with C-reactive protein and 28 joint counts; SDAI, simplified disease activity index; CDAI, clinical disease activity index.
