# Supplementary material for: The comparative responsiveness of Hospital Universitario Princesa Index and other composite indices for assessing rheumatoid arthritis activity
Source: PLoS One. 2019 Apr 10;14(4):e0214717. doi: 10.1371/journal.pone.0214717 (PMC6457549; doi:10.1371/journal.pone.0214717)
Supplement: S4 Table — (DOCX) [file pone.0214717.s007.docx]

**S4 Table.** **Standardized size effects (95% confidence interval) of changes in disease activity assessed with HUPI and several other commonly used to assess response in Rheumatoid Arthritis.**

|  | ACT-RAY | | | PROAR | | EMECAR | |
| --- | --- | --- | --- | --- | --- | --- | --- |
|  | w12  (n=491) | w24 (n=475) | w52 (n=340) | w24  (n=136) | w52 (n=129) | Y2  (n=560) | Y4  (n=440) |
| **HUPI** | 2.30  (2.15-2.46) | 3.13  ((2.95-3.31) | 3.69  (3.48-3.90) | 1.55  (1.30-1.79) | 1.63  (1.37-1.88) | 0.22  (0.11-0.32) | 0.38  (0.27-0.50) |
| **GDA-Pat** | 1.19  (1.06-1.32) | 1.66  (1.52-1.80) | 2.07  (1.91-2.22) | 1.09  (0.86-1.32) | 1.19  (0.95-1.43) | 0.02  (-0.09-0.12) | 0.02  (-0.09-0.13) |
| **GDA-Phy** | 1.86  (1.72-2.00) | 2.46  (2.30-2.62) | 2.74  (2.57-2.92) | 1.23  (1.00-1.47) | 1.40  (1.16-1.64) |  |  |
| **DAS28_ESR** | 2.18  (2.03-2.33) | 2.93  (2.76-3.10) | 3.45  (3.25-3.65) | 1.48  (1.23-1.73) | 1.61  (1.35-1.87) | 0.18  (0.08-0.29) | 0.30  (0.19-0.42) |
| **DAS28-CRP** | 2.20  (2.05-2.35) | 2.85  (2.68-3.03) | 3.28  (3.08-3.48) | 1.47  (1.22-1.73) | 1.63  (1.37-1.89) |  |  |
| **SDAI** | 1.99  (1.84-2.13) | 2.56  (2.40-2.73) | 2.87  (2.68-3.06) | 1.42  (1.17-1.67) | 1.46  (1.20-1.71) |  |  |
| **CDAI** | 1.67  (1.53-1.81) | 2.27  (2.12-2.43) | 2.65  (2.47-2.82) | 1.45  (1.21-1.70) | 1.48  (1.23-1.73) |  |  |

W: week; Y: year; HUPI, *Hospital Universitario La Princesa* Index; GDA-Pat: global disease assessment by patient; GDA-Phy: global disease assessment by physician; DAS28, disease activity score calculated with erythrocyte sedimentation rate and 28 joint counts; DAS28-CRP, disease activity score calculated with C-reactive protein and 28 joint counts; SDAI, simplified disease activity index; CDAI, clinical disease activity index.
