# Supplementary material for: The comparative responsiveness of Hospital Universitario Princesa Index and other composite indices for assessing rheumatoid arthritis activity
Source: PLoS One. 2019 Apr 10;14(4):e0214717. doi: 10.1371/journal.pone.0214717 (PMC6457549; doi:10.1371/journal.pone.0214717)
Supplement: S5 Table — (DOCX) [file pone.0214717.s008.docx]

**S5 Table**. Characteristics of patients with opposite classification with EULAR and HUPI response criteria.

|  | **Tender 28JC** | | **Swollen 28JC** | | **GDA-Pat** | | **GDA-Phy** | | **ESR** | | **CRP** | | **Response** | |
| --- | --- | --- | --- | --- | --- | --- | --- | --- | --- | --- | --- | --- | --- | --- |
| **Week** | **0** | **12** | **0** | **12** | **0** | **12** | **0** | **12** | **0** | **12** | **0** | **12** | **EULAR** | **HUPI** |
| **PT#1** | 11 | 4 | 5 | 5 | 70 | 43 | 42 | 37 | 6 | 3 | 7.8 | 1.6 | good | none |
| **PT#2** | 17 | 5 | 11 | 2 | 68 | 63 | 62 | 13 | 2 | 1 | 6.0 | 14.1 | good | none |
| **PT#3** | 14 | 6 | 2 | 6 | 49 | 12 | 51 | 18 | 8 | 4 | 0.2 | 0.2 | good | none |
| **PT#4** | 22 | 28 | 3 | 2 | 84 | 28 | 41 | 15 | 26 | 12 | 2.3 | 0.4 | none | good |

JC: joint count; GDA-Pat: global disease assessment by patient; GDA-Phy: global disease assessment by physician; ESR: erythrocyte sedimentation rate; CRP-C-reactive protein; PT: patient.
