# Supplementary material for: The comparative responsiveness of Hospital Universitario Princesa Index and other composite indices for assessing rheumatoid arthritis activity
Source: PLoS One. 2019 Apr 10;14(4):e0214717. doi: 10.1371/journal.pone.0214717 (PMC6457549; doi:10.1371/journal.pone.0214717)
Supplement: S6 Table — (DOCX) [file pone.0214717.s009.docx]

**S6 Table. Accuracy of EULAR-RC and HUPI-RC assessed by their correlation with change in GDA-Phy between baseline and different visits of ACT-RAY**

|  |  | β coefficient | 95% C.I. | P | Akaike IC |
| --- | --- | --- | --- | --- | --- |
| **Week 12** | EULAR response  None  Moderate  Good  Constant | Reference  11.7  19.6  18.2 | -  5.5 – 18.0  13.2 – 26.0  12.4 – 24.0 | -  <0.001  <0.001  <0.001 | 8.81 |
|  | HUPI response  None  Moderate  Good  Constant | Reference  12.1  19.4  19.7 | -  7.7 – 16.5  14.9 – 24.0  16.1 – 23.3 | -  <0.001  <0.001  <0.001 | 8.76 |
| **Week 24** | EULAR response  None  Moderate  Good  Constant | Reference  21.3  32.1  12.1 | -  10.5 – 32.0  21.5 – 42.7  1.8 – 22.5 | -  <0.001  <0.001  0.022 | 8.74 |
|  | HUPI response  None  Moderate  Good  Constant | Reference  10.3  25.2  21.2 | -  4.2 – 16.4  19.5 – 30.8  15.9 – 26.5 | -  0.001  <0.001  <0.001 | 8.65 |
| **Week 52** | EULAR response  None  Moderate  Good  Constant | Reference  29.0  40.4  8.3 | -  13.4 – 44.6  25.2 – 55.5  -6.7 – 23.3 | -  <0.001  <0.001  0.279 | 8.86 |
|  | HUPI response  None  Moderate  Good  Constant | Reference  13.7  27.6  22.2 | -  4.9 – 22.5  19.7 – 35.5  14.6 – 29.8 | -  0.002  <0.001  <0.001 | 8.81 |

Abbreviations: GDA-Phy, global disease assessment by physician; C.I., confidence interval; p, p-value; Akaike IC, Akaike information criteria.

Constant’s value represent average ∆GDA-Phy for “None” response. β coefficients for “Moderate” and “Good” responses represent the average increase in ∆GDA-Phy with respect to the constant value (“None” considered reference).
