# Supplementary material for: The comparative responsiveness of Hospital Universitario Princesa Index and other composite indices for assessing rheumatoid arthritis activity
Source: PLoS One. 2019 Apr 10;14(4):e0214717. doi: 10.1371/journal.pone.0214717 (PMC6457549; doi:10.1371/journal.pone.0214717)
Supplement: S7 Table — (DOCX) [file pone.0214717.s010.docx]

**S7 Table. Accuracy of EULAR and HUPI-based response criteria assessed by their correlation with ∆GDA-Phy in PROAR and ∆GDA-Pat in EMECAR.**

|  |  | **β coefficient** | **95% CI** | **p** | **Akaike IC** |
| --- | --- | --- | --- | --- | --- |
| **PROAR**  **Month 6** | **EULAR response**  **None**  **Moderate**  **Good**  **Constant** | Reference  26.5  36.3  2.3 | -  18.0 – 35.0  13.2 – 26.0  -4.5 – 9.1 | -  <0.001  <0.001  0.502 | 8.93 |
|  | **HUPI response**  **None**  **Moderate**  **Good**  **Constant** | Reference  18.5  34.9  7.0 | -  7.7 – 16.5  14.9 – 24.0  1.2 – 12.7 | -  <0.001  <0.001  0.018 | 8.76 |
| **PROAR**  **Month 12** | **EULAR response**  **None**  **Moderate**  **Good**  **Constant** | Reference  34.6  42.6  -1.5 | -  25.0 – 44.2  32.7 – 52.4  -9.4 – 6.4 | -  <0.001  <0.001  0.706 | 9.01 |
|  | **HUPI response**  **None**  **Moderate**  **Good**  **Constant** | Reference  20.0  35.7  7.3 | -  9.8 – 30.1  27.0 – 44.5  15.9 – 26.5 | -  0.001  <0.001  0.040 | 9.06 |
| **EMECAR**  **Year 4** | **EULAR response**  **None**  **Moderate**  **Good**  **Constant** | Reference  15.6  25.1  -8.5 | -  10.5 – 20.6  18.5 – 31.8  -11.4 – -5.2 | -  <0.001  <0.001  <0.001 | 9.17 |
|  | **HUPI response**  **None**  **Moderate**  **Good**  **Constant** | Reference  15.7  27.9  -7.0 | -  10.3 – 21.1  20.5 – 35.3  -9.7 – -4.4 | -  <0.001  <0.001  <0.001 | 9.16 |

Abbreviations: GDA-Phy, global disease assessment by physician; C.I., confidence interval; p, p-value; Akaike IC, Akaike information criteria.
Constant’s value represent average ∆GDA-Phy for “None” response. β coefficients for “Moderate” and “Good” responses represent the average increase in ∆GDA-Phy with respect to the constant value (“None” considered reference).
